# Hyperadherence of *Pseudomonas taiwanensis* VLB120ΔC increases productivity of (*S*)‐styrene oxide formation

**DOI:** 10.1111/1751-7915.12378

**Published:** 2016-07-14

**Authors:** Karolin Schmutzler, Katharina Kupitz, Andreas Schmid, Katja Buehler

**Affiliations:** ^1^ Laboratory of Chemical BiotechnologyDepartment of Biochemical and Chemical EngineeringTU Dortmund UniversityEmil‐Figge‐Strasse 6644227DortmundGermany; ^2^ Department of Solar MaterialsHelmholtz‐Centre for Environmental Research ‐ UFZPermoserstrasse 1504318LeipzigGermany

## Abstract

The attachment strength of biofilm microbes is responsible for the adherence of the cells to surfaces and thus is a critical parameter in biofilm processes. In tubular microreactors, aqueous‐air segmented flow ensures an optimal oxygen supply and prevents excessive biofilm growth. However, organisms growing in these systems depend on an adaptation phase of several days, before mature and strong biofilms can develop. This is due to strong interfacial forces. In this study, a hyperadherent mutant of *Pseudomonas taiwanensis* VLB120ΔCeGFP possessing an engineered cyclic diguanylate metabolism, was applied to a continuous biofilm process for the production of (*S*)‐styrene oxide. Cells of the mutant *P*. * taiwanensis* VLB120ΔCeGFP Δ04710, showing the same specific activity as the wild type, adhered substantially stronger to the substratum. Adaptation to the high interfacial forces was not necessary in these cases. Thereby, 40% higher final product concentrations were achieved and the maximal volumetric productivity of the parent strain was significantly surpassed by *P. taiwanensis* VLB120ΔCeGFP Δ04710. Applying mutants with strong adhesion in biofilm‐based catalysis opens the door to biological process control in future applications of catalytic biofilms using other industrially relevant strains.

## Introduction

Biofilms are densely packed microbial communities of cells enclosed in extracellular polymeric substances (EPS) adherent to the interphase of aqueous systems (Flemming *et al*., 2007). They attracted increasing attention as self‐immobilized biocatalysts for the production of industrially relevant chemicals since they feature increased resistance to toxic chemicals and physical robustness compared with their planktonic counterparts (Rosche *et al*., 2009; Halan *et al*., 2012). Besides numerous examples for the production of bulk chemicals like ethanol (Weuster‐Botz *et al*., 1993; Kunduru and Pometto, 1996), lactic acid (Ho *et al*., 1997; Tay and Yang, 2002) or butanol (Qureshi *et al*., 2004), biofilms were also used for the synthesis of fine chemicals (Hekmat *et al*., 2003; Li *et al*., 2006; Gross *et al*., 2007; Meleigy and Khalaf, 2009) or enzymes (Govender *et al*., 2010). In the last decades, a variety of different biofilm reactor types has been developed including packed bed reactors, fluidized bed reactors, air lift reactors, up‐flow anaerobic sludge blanket reactors and membrane‐aerated biofilm reactors (Halan *et al*., 2012). Continuous membrane microreactors offer the advantage of high surface to volume ratios which is a key parameter for obtaining high volumetric productivities (Van Loosdrecht and Heijnen, 1993). Recently, the problems of oxygen limitation and clogging which frequently occur in membrane microreactors, were overcome by the introduction of an aqueous‐air segmented flow (Karande *et al*., 2014). The concept of the segmented flow was successfully applied to *Pseudomonas taiwanensis* VLB120ΔC biofilms for the continuous production of (*S*)‐styrene oxide reaching volumetric productivities up to 46 g L_aq_^−1^ day^−1^. However, *P. taiwanensis* VLB120ΔC had to be adapted stepwise to the high shear stresses introduced by the segmented flow, in order to allow the development of a mature biofilm. This adaptation phase was characterized by low volumetric productivities for several days due to the small amount of biomass in the system.

In this work, the problem of weak initial attachment forces was addressed by applying a hyperadherent mutant of the (*S*)‐styrene oxide production strain *P. taiwanensis* VLB120ΔCeGFP. The variant *P. taiwanensis* VLB120ΔCeGFP Δ04710 possessing a genetically engineered cyclic diguanylate (c‐di‐GMP) metabolism (Schmutzler *et al*., 2015, 2016) carried a deletion of a phosphodiesterase highly similar to BifA of other pseudomonads (Kuchma *et al*., 2007, 2010; Jiménez‐Fernández *et al*., 2015). This mutant developed macroscopic aggregates in dependency of the supplied carbon source. In minimal medium supplemented with carbohydrates or gluconate the variant showed heavy autoaggregation with enhanced adhesiveness of the cells due to excreted EPS material and/or enhanced cell surface hydrophobicity compared with the parent strain (Schmutzler *et al*., 2015, 2016). The attachment behaviour of the autoaggregates to silicone rubber and the biofilm formation of *P. taiwanensis* VLB120ΔCeGFP Δ04710 were studied in detail. Mutant and parent strain were cultivated according to Karande *et al*. (2014) under process conditions for the continuous synthesis of (*S*)‐styrene oxide. By applying the hyperadherent mutant, the unproductive adaptation phase of the parent strain was circumvented and the final product concentration was substantially increased.

## Results

### Significantly stronger adhesion of *P. taiwanensis* VLB120ΔCeGFP Δ04710

**Figure 1:**
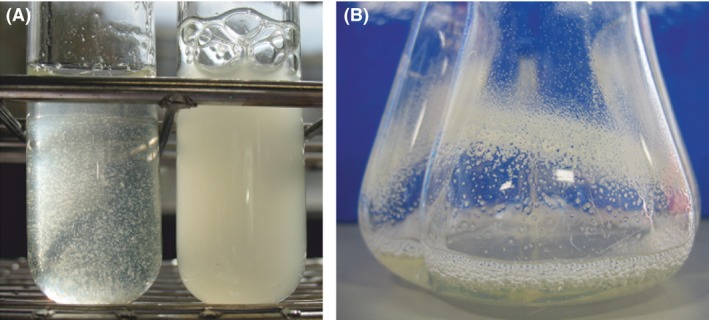
Aggregate formation of *Pseudomonas taiwanensis* VLB120ΔCeGFP Δ04710 in planktonic overnight cultures. A. Autoaggregates of *P. taiwanensis* VLB120ΔCeGFP Δ04710 (left) in comparison with free swimming cells of *P. taiwanensis* VLB120ΔCeGFP (right); strains were grown in M9 minimal medium (0.5% (wt/vol) glucose). B. Adhesion of *P. taiwanensis* VLB120ΔCeGFP Δ04710 to the glass wall of a shaking flask.

**Figure 2:**
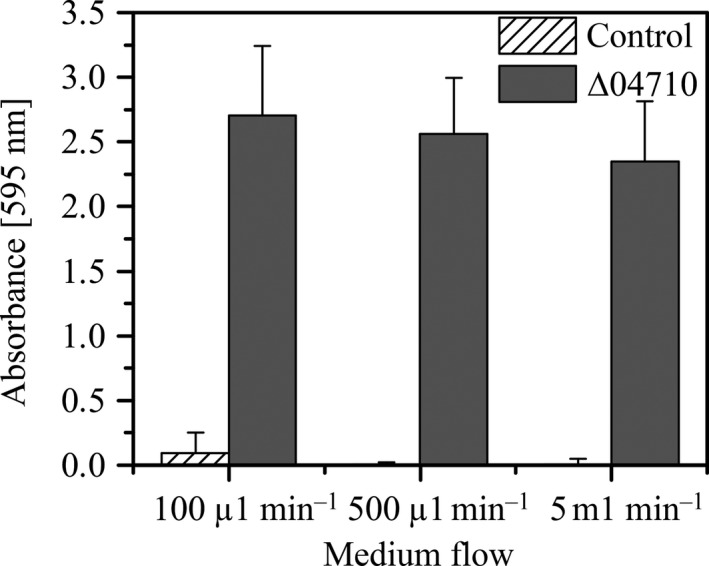
Initial adherence of *Pseudomonas taiwanensis* VLB120ΔCeGFP Δ04710 and control strain *P. taiwanensis* VLB120ΔeGFP to 10 cm long silicone tubings under various flow rates. After 2 h attachment phase, the medium flow of 100 μl min^−1^, 500 μl min^−1^ or 5 ml min^−1^, respectively, was started for 1 h to flush out non‐adhered cells. Afterwards, the silicone tubes were removed and the first 10 cm of each were used for quantification of the attached biomass by crystal violet (CV) staining.

Our previous study showed that *P. taiwanensis* VLB120ΔCeGFP Δ04710 forms cell aggregates (Fig. 1A) containing considerable amounts of EPS, when grown in M9 (0.5% (wt/vol) glucose) medium (Schmutzler *et al*., 2015, 2016). These cell autoaggregates were observed to adhere strongly to polymeric and glass surfaces (Fig. 1B) indicating altered attachment properties of *P. taiwanensis* VLB120ΔCeGFP Δ04710 to solid surfaces. The adhesion behaviour of this mutant to silicone tubing was investigated in more detail as this material was used for the reaction compartment in a biofilm reactor in previous studies (Gross *et al*., 2007, 2010; Karande *et al*., 2014). The impact of different growth rates during biofilm formation can be neglected due to the short assay time of 3 h. The biomass of *P. taiwanensis* VLB120ΔCeGFP Δ04710 adhering to the inner wall of the tubes was substantially higher under all flow conditions compared with the control and decreased only slightly with increasing flow (Fig. 2). In contrast, *P. taiwanensis* VLB120ΔCeGFP showed only negligible amounts of biomass which indicates that the majority of cells were flushed out and only single cells remained attached to the surface. Whether the strong adhesion of *P. taiwanensis* VLB120ΔCeGFP Δ04710 was caused by the altered surface properties of the strain or the excreted EPS of the aggregates needs to be further elucidated.

To make sure that the enhanced attached biomass was not due to higher cell numbers in the *P. taiwanensis* VLB120ΔCeGFP Δ04710 preculture, the remaining inoculum of both strains was centrifuged, dried and quantified. The biomass of the *P. taiwanensis* VLB120ΔCeGFP Δ04710 inoculum was always slightly below the values of the parent strain.

### Hyperbiofilm formation of the mutant *P. taiwanensis* VLB120ΔCeGFP Δ04710

The attachment assay clearly showed an enhanced adhesiveness of *P. taiwanensis* VLB120ΔCeGFP Δ04710 aggregates to silicone surfaces. Therefore, we focused on the impact of the mutation on later stages of biofilm maturation. The biofilm formation of *P. taiwanensis* VLB120ΔCeGFP Δ04710 and, for comparison, the parent strain *P. taiwanensis* VLB120ΔCeGFP were studied in silicone tubings under different flow regimes. An aqueous‐air segmented flow (100 μl min^−1^ medium; 100 μl min^−1^ air) was included.

**Figure 3:**
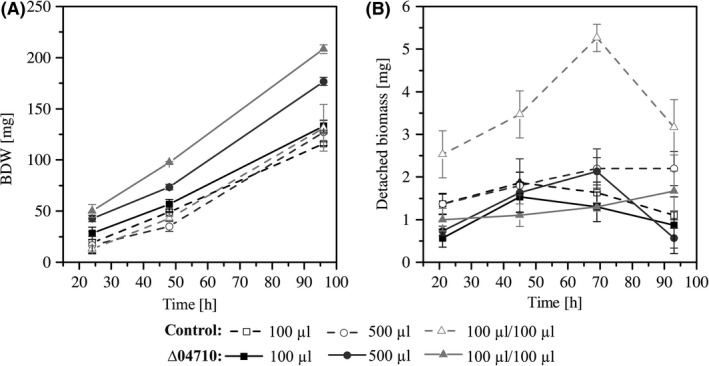
Attached and detached biofilm biomass of *Pseudomonas taiwanensis* VLB120ΔCeGFP Δ04710 and the control strain *P. taiwanensis* VLB120ΔCeGFP grown in silicone tubes applying single‐phase aqueous flow rates of 100 μl min^−1^ and 500 μl min^−1^ or two‐phase aqueous‐air segmented flow of 100 μl min^−1^ medium and 100 μl min^−1^ air. Errors bars represent standard deviation of triplicates. A. Total biofilm dry weight (BDW) of biofilms grown in 40 cm long silicone tubings. B. Detached biofilm biomass during continuous biofilm cultivation. One data point reflects the amount of biomass in 10 ml effluent.

**Figure 4:**
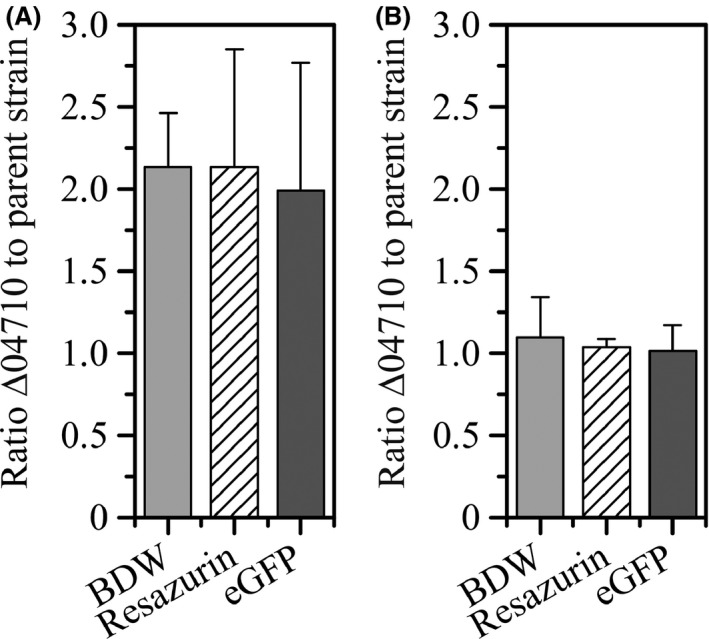
Biofilm dry weight (BDW) and viable cells in biofilms determined via the resazurin and eGFP assay. Values are given as a ratio of the quantities of the mutant *Pseudomonas taiwanensis* VLB120ΔCeGFP Δ04710 to the parent strain *P. taiwanensis* VLB120ΔCeGFP. A. Biofilms after 48 h of standard cultivation under segmented flow conditions. Errors bars represent standard deviation of triplicates. B. Biofilms after 336 h of biotransformation under segmented flow conditions. Error bars represent standard deviation of five replicates.

*Pseudomonas taiwanensis* VLB120ΔC eGFP Δ04710 developed substantially more biofilm biomass under all tested conditions (Fig. 3A). The strongest impact of the mutation was observed in the presence of segmented aqueous‐air flow. In addition, the effluent of *P. taiwanensis* VLB120ΔCeGFP Δ04710 contained less detached biomass compared with the control despite the significant amount of biofilm biomass in the tubings (Fig. 3B). This finding indicated that, besides the increased initial attachment of the mutant, the adhesion of the cells during the biofilm maturation phase was also enhanced compared with the parent strain, which is a highly promising finding. Furthermore, quantification of viable cells (detected via the resofurin assay and eGFP measurement) and total biofilm biomass in *P. taiwanensis* VLB120ΔCeGFP Δ04710 and the parent strain under segmented flow conditions revealed the same ratio of biofilm biomass to viable cells in both, mutant and parent strain biofilms (Fig. 4A). These results clearly emphasize that the enhanced biofilm biomass of *P. taiwanensis* VLB120ΔCeGFP Δ04710 was not solely attributed to an increased EPS formation. Thus, a true benefit of the mutation for biotransformation performance was to be expected due to higher biocatalyst quantities.

### Improving process performance by reactor optimization

In a recent study, *P. taiwanensis* VLB120ΔC biofilm was applied to a tubular microreactor setup under segmented flow conditions (Karande *et al*., 2014). The changed flow conditions led to an altered biofilm architecture, oxygen supply, a better mass transfer of nutrients, substrate and products and finally to an improved biofilm process. Nevertheless, this approach necessitated a step‐wise adaptation of the strain to the higher interfacial forces over 7 days. Thus, the applicability of the hyperadherent *P. taiwanensis* VLB120ΔCeGFP Δ04710 to a microreactor system as described in Karande *et al*. (2014) was elucidated in detail. In order to maximize overall reactor performance, a couple of parameters were modified namely the fluidic conditions (aqueous and air flow rates), tube length, thickness and substrate exposure (Table 1).

**Table 1:** Maximal volumetric productivities in various tubular reactor setups within 120 h biofilm process using *Pseudomonas taiwanensis* VLB120ΔCeGFP Δ04710. The dilution rate was calculated based on the total air and medium flow. In brackets, values are given solely for the medium flow. The maximal volumetric productivities were calculated as an average of at least two replicates

No.	ID (mm)	Wall thickn. (mm)	Length (cm)	Medium flow (μl min^−1^)	Air flow (μl min^−1^)	Dilution rate (h^−1^)	Vol. styrene (ml)	Max. vol. productivity within 120 h (g L_aq_^−1^ day^−1^)	Problems during biofilm process
A	3	1	40	100	100	4.2 (2.1)	20	6.5	
B	3	1	40	250	1000	26.5 (5.3)	20	38.9	Tube connector clogging
C	3	1	40	250	1000	26.5 (5.3)	80	29.5	Tube connector clogging; detachment of biofilm parts
D	3	1	100	250	1000	10.6 (2.1)	20	8.5	Tube connector clogging; tubes kinked
E	3	1	40	250	2000	47.8 (5.3)	20	34.6	
F	2	0.3	200	250	2000	21.5 (2.4)	80	76.6	Detachment of biofilm parts; tubes broke occasionally; tubes kinked
G	2	0.3	40	250	2000	107.4 (11.9)	20	164.0	Tubes broke occasionally
H	1.5	0.75	40	250	2000	191.0 (21.2)	20	92.0	

Low medium and air flow of 100 μl min^−1^ correlated with a low volumetric productivity (Table 1A), mainly due to oxygen and nutrient limitations. Therefore, the medium and air flow was increased to 250 μl min^−1^ and 1 or 2 ml min^−1^ respectively. The change in air flow from 1 to 2 ml min^−1^ prevented clogging of the system occurring at the tube connectors without significant changes in the volumetric productivities (Table 1B and E). Thus, the optimal conditions turned out to be air supply of 2 ml min^−1^ and a tube length of 40 cm due to easier handling of the overall system. Extension of the tube to 1 m (Table 1D) or 2 m (Table 1F) was tested for two conditions but frequently resulted in tube kinking followed by flow blockage. Furthermore, complete submersion of silicone tube in the styrene phase for maximizing styrene mass transfer was also tested for two conditions (Table 1C and F). In both cases, complete submersion did not enhance volumetric productivity, as major parts of the biofilms were infrequently detached, probably due to toxification. Therefore, a partly submerged setup was preferred. Higher volumetric productivities could be achieved by applying tubes with smaller ID and thinner walls (Table 1F and G) due to better mass transfer of styrene and (*S*)‐styrene oxide. However, the thinnest tubing (wall thickness 0.3 mm) was very fragile and difficult to handle. Based on several sets of experiments (Table 1A–G), the optimal reactor configuration and running conditions for further biotransformations were as follows: silicone tubing with dimension of 1.5 mm inner diameter (ID), 3 mm outer diameter (OD) and 40 cm length; flow rate 250 μl min^−1^ medium and 2 ml min^−1^ air; and addition of 20 ml of styrene to the reaction compartment as organic substrate and product extraction phase.

### Strong adhesion of *P. taiwanensis* VLB120ΔCeGFP Δ04710 overcomes adaptation phase

Applying the optimal conditions determined in the previous experiments, the biocatalytic performance of *P. taiwanensis* VLB120ΔCeGFP Δ04710 and the parent strain was monitored over 336 h. (*S*)‐styrene oxide accumulation in the organic phase of the reactor was quantified. The organic phase was not exchanged during the whole experiment as (*S*)‐styrene oxide concentrations below 500 mM are nontoxic for *P. taiwanensis* VLB120ΔC biofilms (Gross *et al*., 2010).

**Figure 5:**
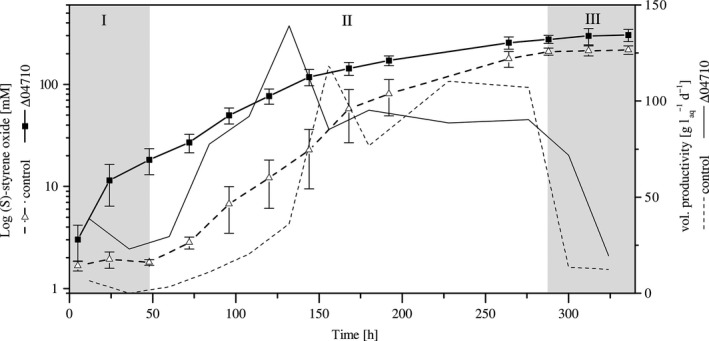
Biotransformation of styrene to (S)‐styrene oxide using *Pseudomonas taiwanensis* VLB120ΔCeGFP Δ04710 and control strain *P. taiwanensis* VLB120ΔCeGFP. The experiment is divided into three phases. Phase I represents the adaptation phase of the control strain while *P. taiwanensis* VLB120ΔCeGFP Δ04710 already produces significant amounts of (S)‐styrene oxide. Phase II shows the production phase of both strains. After 288 h (phase III) the air flow was increased to 4 ml min^−1^. The volumetric productivities are averaged values of five replicates. Error bars represent standard deviation of five independent replicates.

The experiment was divided into three distinct phases (Fig. 5). In phase I, *P. taiwanensis* VLB120ΔCeGFP Δ04710 directly started to produce (*S*)‐styrene oxide after styrene addition with a maximal volumetric productivity of 38.7 g L_aq_^−1^ day^−1^. In contrast, the control strain produced only marginal amounts during the first 48 h. In phase II, the volumetric productivity of both strains further increased to a maximum of 139.1 g L_aq_^−1^ day^−1^ for *P. taiwanensis* VLB120ΔCeGFP Δ04710 after 132 h and 118.4 g L_aq_^−1^ day^−1^ for the parent strain after 156 h. After that time period, both strains showed comparable volumetric productivities fluctuating around 100 g L_aq_^−1^ day^−1^. Finally in phase III, the air flow was increased to 4 ml min^−1^ which directly resulted in a severe decline in the product formation rate. It could be speculated that due to the high flow rates, the respective residence times of the reactants were too short for sufficient conversion. After 336 h of biotransformation, the parent strain had accumulated 217.6 mM of the product while the mutant produced 40% more (*S*)‐styrene oxide (303.6 mM). At all times, (*S*)‐styrene oxide concentrations measured for *P. taiwanensis* VLB120ΔCeGFP Δ04710 were significantly higher (*P* ≤ 0.005) as compared with the parent strain (Fig. 5). The amount of glucose in the effluent remained above 1.5 g l^−1^ which indicates that the system was not glucose limited. The fraction of viable cells determined at the end of the experiment was comparable for both strains (Fig. 4B). Both strains also showed comparable volumetric productivities at later time points of the biotransformation. This indicates that the parent strain and the mutant *P. taiwanensis* VLB120ΔCeGFP Δ04710 exhibited the same specific activity described as the specific rate of (*S*)‐styrene oxide formation per cell (Meyer *et al*., 2006).

## Discussion

### Factors involved in the adhesion of microorganisms

The attachment of bacterial cells to surfaces is the first step during biofilm formation. It is influenced by various parameters including environmental condition, cellular surface structures, hydrophobic interactions and exopolysaccharides (Goulter *et al*., 2009; Petrova and Sauer, 2012). In the present study, the autoaggregating strain *P. taiwanensis* VLB120ΔCeGFP Δ04710 adhered distinctly stronger to the attachment surface compared with the parent strain as shown with the attachment assay and during standard biofilm cultivations. This hyperadhesive behaviour reduced the usual *lag* phases for biofilm catalytic performance in silicone‐based microreactors to zero. In contrast, the parent *P. taiwanensis* VLB120ΔCeGFP showed much weaker adhesion to the tubings and responded to high interfacial forces generated by a two‐phase aqueous‐air flow with an adaptation phase without product formation. In the segmented flow system, the interfacial forces were described to be at least 10^4^‐fold higher than the adhesion forces holding the freshly adhered single cells to the substratum. This consequently resulted in detachment of cells upon segmented flow (Karande *et al*., 2014). On the other hand it was speculated that the secretion of EPS increases the attachment forces of the cells which finally allows the cells to overcome the interfacial forces generated by the segmented flow and form a biofilm. These suggestions are in good agreement to our findings since the aggregates of *P. taiwanensis* VLB120ΔCeGFP Δ04710 were shown via confocal laser‐scanning microscopy and scanning electron microscopy to excrete considerable amounts of EPS material (Schmutzler *et al*., 2015, 2016). Therefore, it can be concluded that *P. taiwanensis* VLB120ΔCeGFP Δ04710 aggregates probably gained additional binding forces due to the presence of EPS as compared with the planktonic cells of the parent strain. In addition to the presence of extracellular polysaccharides, the *P. taiwanensis* VLB120ΔCeGFP Δ04710 mutant exhibited an increased cell surface hydrophobicity compared with the parent strain (Schmutzler *et al*., 2015, 2016). A positive correlation between hydrophobicity and adhesion was described for a variety of other microorganisms (Perez *et al*., 1998; Del Re *et al*., 2000; Pan *et al*., 2006). The fact that a minor fraction of the control strain *P. taiwanensis* VLB120ΔCeGFP survived the start of the segmented flow serving as ‘starters’ for a new, adapted biofilm might be explained by the adhesion of single cells to small uneven places on the silicone surface exhibiting lower hydrodynamic forces. Alternatively, single cells might exhibit a natural variance in the strength of their attachment. Afterwards, these single cells adapted to the flow conditions by the excretion of EPS; a process that lasted in this study approximately 2 days.

Our approach clearly demonstrated that the application of a mutant with strong attachment and increased biofilm formation optimizes the biofilm processes in tubular setups. Similar strategies using mutants with enhanced adhesion during biofilm biocatalysis, are, to our knowledge, only described for strains with overexpressed extracellular adhesins, namely the bacterial fibre protein AtaA in *Acinetobacter* sp. ST‐550 producing indigo (Ishikawa *et al*., 2014) or adhesive curli in *E. coli* K12 producing 5‐halotryptophan (Tsoligkas *et al*., 2011; Perni *et al*., 2013). In the current study, a mutant with a deletion of a gene encoding a putative phosphodiesterase responsible for the degradation of the second messenger c‐di‐GMP was applied (Roemling *et al*., 2013). Mutants of *P. aeruginosa* and *P. putida* carrying deletions of *bifA*, which is highly similar to *PVLB_04710*, also exhibited increased biofilm formation but showed no trophic autoaggregation (Kuchma *et al*., 2007, 2010; Jiménez‐Fernández *et al*., 2015). Mutants showing enhanced biofilm formation due to a mutation regarding phosphodiesterase‐encoding genes are described for numerous other microorganisms including *P. fluorescens*,* E. coli*,* Zymomonas mobilis*,* Clostridium difficile* or *Klebsiella pneumonia* (Gjermansen *et al*., 2006; Starkey *et al*., 2009; Bordeleau *et al*., 2011; Newell *et al*., 2011; Sanchez‐Torres *et al*., 2011; Wilksch *et al*., 2011; Jeon *et al*., 2012). Besides increasing intracellular c‐di‐GMP concentrations, several other strategies are described in literature to enhance the adhesion of bacteria, for instance, by overexpressing adhesive surface proteins or EPS polysaccharides (Ma *et al*., 2006; Ulett *et al*., 2007; Saldaña *et al*., 2009; Borlee *et al*., 2010). Thus, the current approach applying a hyperbiofilm mutant in order to enhance the catalytic performance could be also transferred to other industrially relevant microorganisms when the respective trigger for hyperadhesion is known.

### Enhancing the performance of catalytic biofilms

Over the last decade, several studies have been accomplished to optimize the catalytic performance of *P. taiwanensis* VLB120ΔC biofilms applying different biofilm reactor setups (Table 2) (Gross *et al*., 2007, 2010; Karande *et al*., 2014). While these studies focused on process and reaction engineering targets (Willrodt *et al*., 2015), the current approach was concentrating on biofilm engineering to improve reactor performance. A hyperadherent mutant was applied and the hitherto highest productivity compared with previous studies was observed (139 g L_aq_^−1^ day^−1^). The mutant directly started to produce (*S*)‐styrene oxide after a short pre‐cultivation time of only 24 h and accumulated 40% more product after 14 days of biotransformation compared with the parent strain. Interestingly, the values for *P. taiwanensis* VLB120ΔCeGFP showing a maximal volumetric productivity of 118 g L_aq_^−1^ day^−1^ were also increased compared with former studies indicating that the here applied conditions are not only beneficial for the mutant but in general is also optimal for this specific conversion using this strain. In a previous study it was reported that the wild type was unable to develop biofilms when the segmented flow was directly applied after the attachment phase (Karande *et al*., 2014). Therefore, the respective system was run with a single‐phase aqueous flow for 3 days before the air flow and the biotransformation was started. In the here reported approach, the segmented flow was directly initiated after inoculation followed by a short pre‐cultivation period of 24 h. Afterwards, the biotransformation was started resulting in highly improved volumetric productivities. We found convincing evidence that the mutant and the parent strain exhibited comparable specific activities since the cell numbers and productivities were similar for both strains in the end of the biotransformation. Therefore, the outperformance of the mutant was probably caused by the enhanced adhesiveness to the substratum resulting in higher cell numbers at the beginning of the biotransformation. It can be speculated that matured biofilms of *P. taiwanensis* VLB120ΔCeGFP Δ04710 also adhere stronger and further studies should investigate the EPS composition of both strains at different time points of biofilm development.

**Table 2:** Comparison of the maximum volumetric productivities of *Pseudomonas taiwanensis* VLB120 strains during the biotransformation of styrene to (*S*)‐styrene oxide in different biofilm reactor setups

Reference	Maximal vol. productivity (g L_aq_^−1^ day^−1^)	Strain	Biofilm substratum and flow conditions
Gross *et al*. (2007)	16	*P. taiwanensis* VLB120ΔC	Partly submerged silicone tubes, single‐phase flow
Gross *et al*. (2010)	70	*P. taiwanensis* VLB120ΔC	Partly submerged silicone tubes, single‐phase flow
Halan *et al*. (2010)	28	*P. taiwanensis* VLB120ΔC	Aerated microporous ceramic unit
Karande *et al*. (2014)	46	*P. taiwanensis* VLB120ΔC	Silicone tubes; aqueous‐air segmented flow
This study	118	*P. taiwanensis* VLB120ΔCeGFP	Silicone tubes; aqueous‐air segmented flow
This study	139	*P. taiwanensis* VLB120ΔCeGFP Δ04710	Silicone tubes; aqueous‐air segmented flow

## Conclusion

The application of the hyperadherent *P. taiwanensis* VLB120ΔCeGFP Δ04710 possessing a genetically engineered c‐di‐GMP metabolism in a capillary microreactor for the production of (*S*)‐styrene oxide allowed for fast flow rates. Its strong adhesion to the capillary material resulted in substantially higher biocatalyst cell numbers in the beginning of the process compared with the parent strain. Therefore, *P. taiwanensis* VLB120ΔCeGFP Δ04710 overcame the usual unproductive adaptation phase and distinctly surpassed the final product concentration and maximal volumetric productivity of the parent strain. The current approach of using mutants with engineered adhesion behaviour has great potential for future biofilm processes using other industrially interesting microorganisms.

## Experimental section

All chemicals used in this study were purchased from Sigma‐Aldrich (Steinheim, Germany), Merck (Schwalbach, Germany) or AppliChem (St. Louis, MO, USA) at the highest purity grade available unless stated otherwise.

### Strains and planktonic culture conditions

The microorganisms used in this report were *P. taiwanensis* VLB120ΔCeGFP and *P. taiwanensis* VLB120ΔCeGFP Δ04710 (Schmutzler *et al*., 2015, 2016). *Pseudomonas taiwanensis* VLB120 is deposited at the German Collection of Microorganisms and Cell Cultures (DSMZ), deposition number: DSM 24711. For pre‐cultures, 5 ml of Lysogeny broth (LB) (Bertani, 1951) was inoculated from −80°C glycerol stocks and incubated overnight at 30°C and 200 r.p.m. (2.5 cm amplitude; Multitron standard shaker Infors HT, Bottmingen, Switzerland). Afterwards, 1 ml of the pre‐culture was centrifuged and washed with sterile 0.9% (wt/vol) NaCl solution to reduce the amount of residual LB medium. The cells were diluted 1:100 (vol/vol) in M9 minimal medium (Sambrook and Russel, 2001) supplemented with 0.5% (wt/vol) glucose and 100 μg ml^−1^ streptomycin. Cultures were subsequently cultivated overnight at 30°C and 200 r.p.m. (2.5 cm amplitude; Multitron standard shaker Infors HT) in baffled shaking flasks (airphase/liquid 10:1 (vol/vol).

### Standard biofilm cultivation

The tubular cultivation setup for biofilm characterization was previously described (Schmutzler *et al*., 2015, 2016). In brief, the biofilms were cultivated in silicone tubings (ID 3 mm, OD 5 mm; VWR, Langenfeld, Germany) connected via Tygon tubings (ID 2.06 mm, OD 3.78 mm; Ismatec, Wertheim, Germany). The assembled system was autoclaved and subsequently conditioned for 1 h with M9 minimal medium supplemented with 0.5% (wt/vol) glucose and 100 μg ml^−1^ streptomycin (flow rate 500 μl min^−1^) before it was inoculated with freshly prepared M9 medium pre‐cultures using syringes. Prior to inoculation, aggregates of the *P. taiwanensis* VLB120ΔCeGFP Δ04710 pre‐culture adhering to the glass of the shaking flasks were detached using a sterile inoculation loop. During inoculation, the medium flow was stopped and the systems were kept idle for 2 h to allow initial attachment of the cells to the inner wall of the silicone tubes. Afterwards, the pumps (multichannel peristaltic pump IPC; Ismatec) were started again. Respective flow rates were indicated as either 100 μl min^−1^ medium flow, 500 μl min^−1^ medium flow or 100 μl min^−1^ medium plus 100 μl min^−1^ air flow.

Effluent samples were regularly collected in order to quantify the detached biomass as well as glucose and gluconate. After the respective incubation period (1, 2, 4 days), the silicone tubes with the biofilms were removed for further characterization.

### Biofilm biotransformation

The reactor setup (Fig. S1) and biofilm cultivation was largely identical to the approach of Karande *et al*. (2014) with slight modifications as briefly outlined below. Silicone tubes of various length, wall thickness and ID as listed in Table 1 were used in order to find optimal conditions. The inoculation of the cultivation tubing was done as described above. After the 2 h attachment phase, the air flow was directly started together with the medium flow. Subsequently, the respective medium and air flow ran for 24 h. Then, the biotransformation substrate styrene (20 or 80 ml) was pipetted into the 100 ml reaction compartment that was afterwards tightly sealed to minimize evaporation of the organic phase. When only 20 ml of styrene was added, special care was taken that about 20% of the cultivation tubing was submerged (Gross *et al*., 2010). Sampling of the organic phase was done through the lids of the reaction compartment using a syringe with a 120 mm long needle. Effluent samples were regularly collected to quantify glucose and gluconate.

### Quantification of detached biofilm biomass in the effluent

During standard biofilm cultivations, detached biofilm biomass was quantified in the effluent in 24 h intervals. Therefore, 10 ml of the efflux liquid was collected on ice and subsequently filtered through a weighted and pre‐dried cellulose acetate filter with pore sizes of 0.22 μm (Sartorius Stedim Biotech, Göttingen, Germany) using a water jet pump. The filters were dried overnight to a constant weight at 85°C before the biomass weight was determined by differential weighting.

### Quantification of biofilm biomass in the silicone tubings

The biomass of the biofilms grown inside the silicone tubes during standard biofilm cultivations was quantified as described previously (Schmutzler *et al*., 2015, 2016). Briefly, the biofilm was completely detached by rigorously rolling a cylinder on the sealed tubes before the biofilm was flushed out in pre‐dried, weighted falcon tubes. After centrifugation (30 min; 4°C; Kendro Heraeus Multifuge, Langenselbold, Germany), the biofilm biomass was carefully separated from the supernatant and dried to a constant weight for 48 h at 85°C. The biofilm dry weight (BDW) was quantified by differential weighting.

For experiments in which the amount of viable cells (see below) was quantified in addition, the biofilm sample was split and only 150 μl of the detached biofilm biomass was filtered through the above described cellulose acetate filters, which were afterwards dried and weighted.

### Quantification of viable cells in the biofilms

The BDW quantification summarizes living and dead cells as well as EPS material. To determine the viable cells in the biofilms, resazurin reduction and eGFP fluorescence was measured. The resazurin assay is based on the reduction of resazurin to resofurin by metabolically active cells (Peeters *et al*., 2008). The biofilm biomass was centrifuged (30 min; Kendro Heraeus Multifuge) and resuspended in 0.9% (wt/vol) NaCl solution. Afterwards, 2 ml of diluted (1:10, 1:100, 1:1000 with 0.9% (wt/vol) NaCl) biofilm sample was supplemented with 20 μl of freshly prepared 0.01% (wt/vol) resazurin solution and incubated in the dark (30°C; 200 r.p.m.; 2.5 cm amplitude; Multitron standard shaker Infors HT). After 90 min, 200 μl was transferred to a microtitre plate (PolySorp, black; VWR, Darmstadt, Germany) and the product resofurin was quantified (Infinite M200; Tecan, Mannedorf, Switzerland) via fluorescence (λ_ex_: 535 nm and λ_em_: 590 nm).

In parallel, the eGFP fluorescence of the biofilm cells which was previously shown to directly reflect the number of viable cells in a sample, was quantified (Lehtinen *et al*., 2004). For determining eGFP fluorescence, various dilutions of the respective biofilm samples were prepared using 0.9% (wt/vol) NaCl solution. Samples were measured in a microtiter plate (PolySorp, black; VWR, Darmstadt) on a microtitre plate reader (Infinite M200; Tecan) at λ_ex_: 488 nm and λ_em_: 525 nm.

Other methods like biomass plating turned out to be a very inaccurate for quantifying cell numbers due to cell aggregation.

### Attachment assay

The setup for the attachment assay was identical to the standard biofilm cultivations. After the 2 h attachment phase, the medium flow of 100 μl min^−1^, 500 μl min^−1^ or 5 ml min^−1^, respectively, was started for 1 h to flush out non‐adhered cells. Afterwards, the silicone tubes were removed and the first 10 cm of each were used for quantification of the attached biomass by crystal violet (CV) staining. Since CV binds to negatively charged molecules, both, cells and EPS, were stained (Peeters *et al*., 2008). To obtain a better exposure of the biomass to the dye, the tubes were sliced lengthwise. Subsequently, they were incubated for 5 min in 0.1% (wt/vol) CV solution (in sterile water) at 50 r.p.m. (2.5 cm amplitude; Multitron standard shaker Infors HT). Unbound CV was removed in three sequential washing steps with tap water before the tubes were dried for 1 h at 65°C. The tubes were destained overnight at 50 r.p.m. (2.5 cm amplitude; Multitron standard shaker Infors HT) in 15 ml of an acetone/ethanol (20:80) mixture in tightly sealed reaction tubes. Afterwards, the absorbance of the destaining solution was measured in a VIS photometer (spectrophotometer Biochrom Libra S12; Biochrom, Cambridge, UK) at 595 nm. As a negative control, tubes that were identically incubated but not inoculated with cells, were always included in the experiments.

### Sample preparation and gas chromatography analysis

The volumetric productivity (g L_aq_^−1^ day^−1^) of the biofilms was defined as the amount of (*S*)‐styrene oxide produced per litre of aqueous phase per 24 h (Halan *et al*., 2010). Since it has been shown that less than 10% of the total (*S*)‐styrene‐oxide was stripped into the aqueous effluent, only the organic phase was analysed (Karande *et al*., 2014). (*S*)‐styrene oxide concentrations were quantified on a Focus gas chromatography (GC) gas chromatograph (Thermo Electron Corporation, Dreieich, Germany) as described previously (Karande *et al*., 2014).

### Sample preparation and high‐performance liquid chromatography analysis

For the quantification of the glucose consumption and gluconate production of the biofilm cells, 2 ml of effluent was regularly collected. For purification, organic solvents were extracted using bis‐(2‐ethylhexyl) phthalate and the aqueous phase was analysed on a LaChrom Elite high‐performance liquid chromatography system (Hitachis High Technologies America, Pleasanton, CA, USA) as described earlier (Gross *et al*., 2010).

### Statistical analysis

The data obtained from the biotransformation experiments were analysed using the Mann–Whitney *U*‐test.

## Supporting information


**Fig. S1.** Capillary‐based cultivation set‐up for *(S)*‐styrene oxide production using *Pseudomonas taiwanensis* VLB120ΔC biofilm as catalyst. The Biofilm is growing inside the capillary, sitting in a bottle which is filled partly with the biotransformation substrate styrene. Styrene enters the capillary via diffusion, is converted by the biofilm to (S)‐styrene oxide, which is then again extracted to the styrene reservoir. The tubing is continuously flushed with M9 medium, supplemented with glucose as carbon source.
