# Supplementary figures and images for: Hyperadherence of Pseudomonas taiwanensis VLB120ΔC increases productivity of (S)‐styrene oxide formation
Source: Microb Biotechnol. 2016 Jul 14;10(4):735–44. doi: 10.1111/1751-7915.12378 (PMC5481534; doi:10.1111/1751-7915.12378)

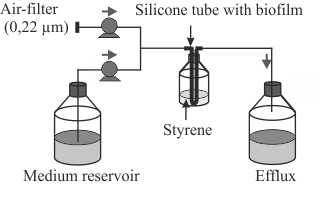

Supplement: Supplementary file 1 — Fig. S1. Capillary‐based cultivation set‐up for (S)‐styrene oxide production using Pseudomonas taiwanensis VLB120ΔC biofilm as catalyst. The Biofilm is growing inside the capillary, sitting in a bottle which is filled partly with the biotransformation substrate styrene. Styrene enters the capillary via diffusion, is converted by the biofilm to (S)‐styrene oxide, which is then again extracted to the styrene reservoir. The tubing is continuously flushed with M9 medium, supplemented with glucose as carbon source. [file MBT2-10-735-s001.png]
